# Book Notices

**Published:** 1856-05

**Authors:** 


### BOOK NOTICES.

*American Journal of Pharmacy.*

This excellent Journal for March, 1856, is received. It is filled, as usual, with valuable and interesting matter. It contains a notice of the new edition of the Pharmacopœia of the United States, which we copy for the benefit of our readers who may not have seen the work:—

"Since the publication of the first Edition of the Pharmacopœia of 1820, the present is the first instance when it has been found necessary to issue an inter-periodical edition. The Committee of Publication have not felt justified in making additions to the work of preparations of recent origin, nor of new drugs, and have limited their labor to the correction of some few errors of the previous edition, and to a few changes (in the processes for Solutions of Nitrate of Iron and Citrate of Magnesia) which seemed to be much needed. In 'accordance with an expressed wish from several respectable sources,' this edition has been published in a cheaper form, so that it is within the reach of all; and it is to be desired that in the future Decennial revisions, attention will be given to this point, so essential in effecting the wide adoption of the Pharmacopœia as the authorized guide book for the Apothecary in the United States. The book is well printed, and the price is but one dollar, the previous edition being $2,50. Every apothecary and physician should obtain a copy of it to be able to tell what are the preparations officially recognized as officinal."

---

*Clinical Lectures on the Diseases of Women and Children:* By GUNNING S. BEDFORD, A.M., M.D., Professor of Obstetrics, the Diseases of Women and Children and Clinical Midwifery in the University of New York. "*Medicus curat morbos, natura sanat.*" Second edition. New York: S. S. & W. Wood, 1856.

From the preface to the second edition, we learn that the first edition was exhausted in a period of three months. He tells us, in the first edition, that these lectures have been reported by Drs. Palmer Woodcock, H. C. Cooper, and Thos. A. Gregory, and that most of them have been published in the *American Lancet.* A medical gentleman, in whose judgment we have great confidence, says that "it is a pity they had not been allowed to remain there," an opinion in which we fully coincide.

J.

---

*On Bandaging and other Operations of Minor Surgery:* By F. W. SARGENT, M.D., Member of the College of Physicians of Philadelphia, &c., &c. New edition—revised and enlarged, with one hundred and eighty illustrations. Philadelphia: Blanchard & Lea, 1856.

The author professes to present information in reference to subjects slightly alluded to in systematic courses of lectures, or in most of the published treatises on the science of surgery, and yet he says that "originality can scarcely be expected in a work of this kind, excepting, perhaps, in its composition." The book contains much that is useful to the young surgeon, but, in our judgment, is open to the same objection that may be urged against all of the compends, abridgements, &c., of the different branches of medicine. They are likely to divert the attention from larger and more complete works, while, at the same time, they only partially supply the wants of the practitioner.

The book is for sale by Keen & Lee, Chicago, Illinois. J.

*How to Nurse Sick Children:* Intended especially as a help to the Nurses at the Hospital for Sick Children, but containing directions which may be found of service to all who have the charge of the Young. New York: S. S. & W. Wood, 1856.

This little book has been laid on our table, and we should be very glad to see it in every sick room in our city, for it is full of useful hints and valuable directions for the management of sick children. J.

---

*A Treatise on Medical Jurisprudence.* By FRANCIS WHARTON, author of a Treatise on American Law, &c., and MORETON STILLE, M.D., Lecturer on the Principles and Practice of Medicine, in the Philadelphia Association for Medical Instruction. Philadelphia, Kay & Brother, 17 and 19 South Fifth Street, 1855.

This work is the joint production of both medicine and law. Of the legal author, we know less than of the medical, although we believe he holds a prominent position in his profession. Dr. Stille, the representative of our own profession, was a young man of rare abilities and of very superior attainments. In his death, which took place on the 20th of August last, the profession sustained a severe loss.

The work is divided into six books, the first of which treats of mental unsoundness; the second, questions relative to the fœtus and new-born child; the third, questions arising out of the difference of sex; the fourth, of questions relative to identity; the fifth, of questions relative to the cause of death; and the sixth, of the legal relations of homicide, fœticide, and infanticide. The second, third, fourth, and fifth books, are by Dr. Stille.

The work is full and complete, so far as the wants of the medical witness is concerned; and we unhesitatingly recommend it to our readers.

For sale by D. B. Cooke & Co., Chicago. J.

---

*Report of the Pennsylvania Hospital for the Insane, for the year* 1855. By THOMAS S. KIRKBRIDE, M.D., Physician to the Institution. Published by order of the Board of Managers. Philadelphia, 1856.

The total number of patients in the hospital during the year, was 399. The highest number at any one time, was 242, the lowest 223; and the average number under treatment, during the whole period, was 233. Of the patients discharged during the year 1855, were

| | |
|---|---|
| Cured, | 101 |
| Much improved, | 13 |
| Improved, | 23 |
| Stationary, | 11 |
| Died, | 21 |
| Total, | 169 |

The report contains valuable statistical tables, presenting a carefully prepared digest of all the cases received into the hospital, from its opening in 1841 to the present time. J.

---

*The Practitioners' Pharmacopœia and Universal Formulary, containing* 2000 *Classified Prescriptions, selected from the practice of the most eminent British and Foreign medical authorities, with an Abstract of three British Pharmacopœias, and much other useful information for the practitioner and student.* By JOHN FOOTE, M.R.C.S.—London. Revised, with corrections and additions, by an American Physician. New York, Samuel S. & William Wood, 1855.

This work is of a class for which we have no partiality. The well-educated physician will combine his remedies to suit the special conditions of individual cases. There is, however, in this little book much that is useful, as will be inferred from its title page. J.

---

*A Practical Hand-Book of Medical Chemistry.* By JOHN E. BOWMAN, F.C.S., Professor of Practical Chemistry in King's College, London. Second American, from the third and revised London edition, with illustrations. Philadelphia, Blanchard & Lea, 1855.

We have received from the publishers a copy of the above work, which we take pleasure in acknowledging, and also in recommending to our readers and to students of medicine. For the benefit of those not acquainted with it, we extract a portion of the preface to the first edition, from which an idea will be gained of its scope, &c.

"The want which, as a teacher of Practical Chemistry in a Medical School, I have long felt, of a small manual containing instructions for the examination and analysis of urine, blood, and a few other of the more important animal products, both healthy and morbid, and comprising also directions for the detection of poisons in organic mixtures and in the tissues, was my chief inducement in undertaking to write the present little work.

"In doing this, my endeavor has been, to supply a book that will be found useful, not only to the Medical Student, but also to the Practitioner, to whom the value and importance of the applications of modern chemistry and microscopic analysis to his art, are becoming daily more and more apparent."

The present edition embodies all the new discoveries and improvements in medical chemistry up to the time of its publication. J.

---

*Tully's Materia Medica and Pharmacology, for January,* 1856, is received. This No. completes 954 pages of the work. It is published at 25 cents per No. by Jefferson Church, M.D., Springfield, Mass.

Some of our readers may wish to know what Dr. Tully says of veratrum viride. It is considered in connection with sanguinaria vernalis and lobelia inflata, in the Proem to the class called by the author Erethistica.

The following is the language of the author:—

"I have repeatedly seen a very obvious, indeed prominent degree of that part of an erethistic operation, on which the primary part of my definition is founded, produced by veratrum viride and sanguinaria, more frequently in children, but repeatedly in adults. I do not feel equally confident of ever having seen them produce those effects, in any decided degree, on which the secondary part of my definition is founded; and yet a priori, I should sooner expect they would produce them than any other. The term acrid-narcotic as employed by authors, has reference as much to such articles as veratrum viride, sanguinaria vernalis, etc., as to any other, in which an individual active proximate principle, as sanguinaria or sanguinarine for example, is at one and the same time acrid, and as is supposed, narcotic. But do veratrum viride and sanguinaria or the active proximate principle of the latter, viz.: sanguinaria or sanguinarine (I do not pretend to say anything of the active proximate principle of the former, whatever it may be, for it has never been isolated, and therefore its nature and character has never been determined) possess any true and genuine narcotic power? Does either veratrum viride or sanguinaria vernalis, ever prove directly antirritant, anodyne, or soporific? Is not such an operation essential to a true and genuine narcotic? Although I once entertained a different opinion, I believe, mainly on the ground of authority, yet I am now obliged to doubt whether either veratrum viride or sanguinaria vernalis, or the active principle of the latter, ever prove directly antirritant, anodyne, or soporific. As now appears to me, their ultimate effects vary very considerably from the ultimate effects of the ordinary true and unequivocal narcotics. But do they ever produce either of the sets of effects which constitute the primary part of my definition of erethistics; or even the set of effects which constitute the secondary part of my definition? At the time that my professional pupils and myself were investigating the powers, operations, and effects of these agents, I had no very definite and precise ideas even of a narcotic power, and much less of an erethistic power; so that none of the observations made at that time, are now available towards settling the present question. But the term acrid-narcotic, I believe, is not unfrequently applied to articles affording much less evidence of a true and genuine narcotic power than sanguinaria vernalis and veratrum viride. Now, the only doubt in regard to veratrum viride and sanguinaria vernalis, for which there seems to me to be the least shadow of ground, is whether they are narcotics and erethistics, or erethistics merely. I have been in the habit of employing both of them ever since the summer of 1810, and I have never witnessed any thing like a direct antirritant, anodyne, or soporific effect from either of them; though I have often witnessed erethism when they have been given in comparatively large doses, at short and regular intervals. For myself I have not the least doubt that they are non-narcotic erethistics; and yet as some professional friends who even recognize the power that I call erethistic, still insist that these articles are narcotic instead of erethistic, I am perfectly willing that my readers should have all the benefit of these doubts, thus having the question fairly brought before them. I have never seen any spasms or convulsions produced by veratrum viride or sanguinaria vernalis; nor have I ever known them destroy life; and with me there is as great a deficiency of definite and precise testimony upon these points as of actual knowledge.

Are veratrum viride and sanguinaria vernalis ever capable of relieving the acinesiæ, parases and paralyses, rheumatalgia and neuralgia vera, or chronic forms and stages of rheumatismus and podagra? I have never known them employed in the acinesiæ, pareses or paralyses, nor in neuralgia vera, but in all the other diseases above-mentioned they have a well established efficacy. As there is unquestionably a strong analogy between the alcaloid now improperly called veratrine, and the active proximate principle of veratrum viride, as well as sanguinarine (the principal difference consisting in the fact that the last two are entirely destitute of all cathartic power, while the first is hydragogue cathartic) I can not see any good reason why veratrum viride and sanguinaria vernalis, but much more especially their active proximate principles in a separate state, might not be serviceable in the acinesiæ, pareses, and paralyses, and perhaps even in neuralgia vera, if employed as is directed for what is called veratrine, and for delphanine. But I have never known them tried in these diseases.

If we should admit that veratrum viride and sanguinaria vernalis are narcotics, it would not follow that lobelia inflata is so likewise. The erethistic power of this latter agent is materially different in quality from the erethistic power of the clematidiæ, anemoneæ, etc. If I were to rank veratrum viride and sanguinaria vernalis with the narcotics, I should not by any means associate lobelia inflata with them. Its utter destitution of any thing even like a true narcotic power will be shown when I come to treat of it individually. Though some varieties of erethistic power may be and are commonly mistaken for a narcotic power, yet some of them are so unlike, as never to be thus confounded with it, by any accurate observer at least. I feel absolutely certain that lobelia inflata is not narcotic; but if I mistake not, when administered in a comparatively large quantity in uniform doses, at regular and very short intervals, it is decidedly erethistic.

I never happened to witness either convulsions or death produced by either of these three articles; but if we may rely on very vague testimony, they extinguish life by occasioning extreme constitutional irritation and rapidly progressive exhaustion of the energies and powers of the nerves of chimical action, nutrition, and reproduction. Such being the fact, I should think that if they ever produce convulsions, they must be of the clonic sort."

